# Genomic and Proteomic Characterization of the Extended-Spectrum β-Lactamase (ESBL)-Producing *Escherichia coli* Strain CCUG 73778: A Virulent, Nosocomial Outbreak Strain

**DOI:** 10.3390/microorganisms8060893

**Published:** 2020-06-13

**Authors:** Daniel Jaén-Luchoro, Antonio Busquets, Roger Karlsson, Francisco Salvà-Serra, Christina Åhrén, Nahid Karami, Edward R. B. Moore

**Affiliations:** 1 Department of Infectious Diseases, Sahlgrenska Academy, University of Gothenburg, 40234 Gothenburg, Sweden; roger.karlsson@nanoxisconsulting.com (R.K.); francisco.salva.serra@gu.se (F.S.-S.); christina.ahren@vgregion.se (C.Å.); nahid.karami@microbio.gu.se (N.K.); erbmoore@ccug.se (E.R.B.M.); 2 Department of Clinical Microbiology, Sahlgrenska University Hospital, Region Västra Götaland, 41346 Gothenburg, Sweden; 3 Culture Collection University of Gothenburg (CCUG), Sahlgrenska Academy of the University of Gothenburg, 41346 Gothenburg, Sweden; 4 Centre for Antibiotic Resistance Research (CARe) at University of Gothenburg, 40530 Gothenburg, Sweden; 5 Microbiology, Department of Biology, University of the Balearic Islands, 07122 Palma de Mallorca, Spain; toni.busquets@uib.es; 6 Nanoxis Consulting AB, 40016 Gothenburg, Sweden; 7 Swedish Strategic Program against Antimicrobial Resistance (Strama), Region Västra Götaland, Gothenburg, Sweden

**Keywords:** *E. coli*, ESBL, β-lactamase, outbreak, CTX-M, antibiotic resistance, *Enterobacteriaceae*, nanopore, genomics, proteomics

## Abstract

*Escherichia coli* strain CCUG 78773 is a virulent extended-spectrum β-lactamase (ESBL)-producing ST131-O25b type strain isolated during an outbreak at a regional university hospital. The complete and closed genome sequence, comprising one chromosome (5,076,638 bp) and six plasmids (1718–161,372 bp), is presented. Characterization of the genomic features detected the presence of 59 potential antibiotic resistance factors, including three prevalent β-lactamases. Several virulence associated elements were determined, mainly related with adherence, invasion, biofilm formation and antiphagocytosis. Twenty-eight putative type II toxin-antitoxin systems were found. The plasmids were characterized, through in silico analyses, confirming the two β-lactamase-encoding plasmids to be conjugative, while the remaining plasmids were mobilizable. BLAST analysis of the plasmid sequences showed high similarity with plasmids in *E. coli* from around the world. Expression of many of the described virulence and AMR factors was confirmed by proteomic analyses, using bottom-up, liquid chromatography-tandem mass spectrometry (LC-MS/MS). The detailed characterization of *E. coli* strain CCUG 78773 provides a reference for the relevance of genetic elements, as well as the characterization of antibiotic resistance and the spread of bacteria harboring ESBL genes in the hospital environment.

## 1. Introduction

Extended-spectrum β-lactamases (ESBLs) are enzymes able to hydrolyze and inactivate clinically-relevant β-lactam antibiotics, such as penicillins, cephalosporins and monobactams [1]. There are several types of ESBL enzymes; among them, CTX-M is one of the most prevalent worldwide [2]. Because genes encoding ESBLs are commonly plasmid-borne, they can be transferred to other strains of the same or different species, contributing to the spread of antibiotic resistance. In fact, propagation of ESBL genes among bacteria has been reported worldwide, representing a global problem for human health [3,4,5].

Members of the family *Enterobacteriaceae*, such as *Escherichia coli*, harboring ESBL genes represent an increasingly important threat for human health [6]. Infections caused by ESBL-producing *E. coli* (ESBL-*E. coli*), or any other ESBL-producing *Enterobacteriaceae*, extend patient morbidity, mortality and length of stay at hospitals, imparting significant economic burdens to the health care system. For these reasons and to prevent transmission of resistance and outbreaks caused by these bacteria, the study and understanding of ESBL-producing bacteria is highly important [7].

Recently, the marked decrease in costs of next-generation sequencing (NGS) technologies has allowed their progressively extensive use in research, as well as implementation in clinical routines [8,9]. In the context of outbreaks of ESBL-producing infectious bacteria, having complete genome sequences as a reference, especially including associated plasmid sequences, advances risk assessment and expands potential for increasing efficacy for treatment. Addressing issues, such as where outbreaks start; whether infection or resistance is facilitated through horizontal transmission and, if yes, are plasmids involved, how many and which ones; as well as how plasmids have spread among strains, is of great benefit [10]. Obtaining complete genome sequences, using only short-read sequencing platforms, such as Illumina, is usually difficult, since complex or repetitive regions often cannot be resolved [11]. However, the combination of Illumina short-reads with long-reads obtained with third-generation platforms, such as the MinION from Oxford Nanopore Technologies, allows compilation of high quality whole-genome sequences in relatively short time [11], enabling the study of genetic elements in their original genomic context, e. g., whether a specific element is chromosome- or plasmid-encoded. When genome sequences are generated, compilations of genomic information regarding antibiotic resistance genes or virulence factors allow comprehensive characterizations of what specific strains can do, potentially. However, it is important to consider also what is actually expressed by performing further characterizations, such as proteomics-based expression analyses. Some genes, such as plasmid-borne β-lactamases, tend to be constitutively expressed [12], i.e., genes are transcribed and then translated into the functional proteins without induction. In such cases, the constitutive expression of the β-lactamases has prepared the bacterium for attack by antibiotics before it is exposed to them, which is highly relevant in a clinical context. Nevertheless, the expression levels of plasmid-borne genes may vary, depending on different genetic signatures of the plasmids or different bacterial hosts [13]. Sometimes, the particular constitutive or inducible expression of a specific gene is not well-known. For that reason, it is important to study which genes are expressed initially, without exposure to any antibiotics, in order to determine what to expect from a specific pathogen, depending on the features encoded by its genome.

Here, we present the complete genome sequence of the ESBL-producing *E. coli* strain CCUG 73778. This bacterium, of sequence type-serotype ST131-O25b, a pandemic, virulent and multi-antibiotic resistant strain, caused septicemia and was an early isolate in a polyclonal outbreak of ESBL-producing bacteria affecting post-surgical, neonatal infants hospitalized at the Sahlgrenska University Hospital (Gothenburg, Sweden) [14,15]. A genomic characterization, focusing on the main features related to antibiotic resistance, virulence factors and toxin-antitoxin systems (TAS), is presented. Furthermore, information is presented about the constitutive expression, obtained by proteomic analysis of *E. coli* strain CCUG 73778, cultured at optimal conditions (liquid broth culture), i.e., with no exposure to antibiotics or other stress.

## 2. Materials and Methods

### 2.1. Strain and Growth Conditions

*Escherichia coli* strain CCUG 73778 (designated as strain E10787 in previous publications) was isolated from the blood of a four month-old infant at the Sahlgrenska University Hospital (Gothenburg, Sweden) in 2008 [14,15], during an outbreak caused by ESBL-producing *E. coli*. At the time of the positive blood culture, the infant was colonized in the gut (stool), nostrils and in a wound, by the same bacterium. The strain was characterized as the ST131-O25b type and was observed to carry the CTX-M-15-encoding gene associated with a plasmid [14,15]. Seventeen months later, the infant was still colonized with ESBL-*E. coli* of the same type and harboring the same plasmid with *bla*_CTX-M-15_ [15]. *E. coli* strain CCUG 73778 was stored at −80 °C; the strain was cultivated at 37 °C, for 24 h, on Blood Agar medium (Columbia Agar Base plus 5% defibrinated horse blood). 

### 2.2. DNA Extraction

One inoculating-loop of strain biomass was taken from fresh, pure cultures and suspended in EDTA-saline buffer. DNA was extracted, for Illumina sequencing, using the Wizard^®^ Genomic DNA Purification Kit (Promega, Madison, WI, USA), and then purified, with the column-based kit, DNA Clean & ConcentratorTM-100 (Zymo Research, Irvine, CA, USA). Another inoculating-loop of fresh strain biomass was taken for Oxford Nanopore sequencing, suspended in EDTA-saline buffer (0.15 M NaCl, 0.01 M EDTA, pH 8.0) and lysed with lysozyme (300 mg/mL) at 37 °C, for 1 h. DNA was extracted, using an established protocol [16], optimized for genome sequencing [17].

### 2.3. DNA Sequencing

Paired-end (short) reads were obtained, using the Illumina MiSeq platform on 2 × 300 mode (Illumina Inc., San Diego, CA, USA). Nanopore sequence (long) reads were obtained, using the MinION device (Oxford Nanopore Technologies Ltd., Oxford, UK). The DNA library was prepared using the Rapid Sequencing Kit (SKQ-RAD003), and then loaded onto a Flow Cell, FLO-MIN106 vR9.4. The sequencing run was performed on a MinION Mk101B device, for 48 h, with MinKNOWN software v1.14.1. Sequence read base-calling was done, using Albacore v2.3.3 (Oxford Nanopore Technologies Ltd., Oxford, UK).

### 2.4. Genome Assembly

Illumina sequence reads were trimmed, using Sickle v1.2 [18], setting a minimal Phred quality score of 30. A hybrid assembly of high-quality Illumina short-reads in combination with Oxford Nanopore long-reads was performed, using SPAdes v3.11.1 [19,20], with the default parameters, including the options *--careful*, *--only-assembly*, *--cov-cutoff auto* and *--nanopore*.

### 2.5. Whole Genome Sequence Quality Control and Annotation

The quality parameters of the assembly were analyzed, using the Quality Assessment Tool for Genome Assemblies (QUAST) v4.5 [21]. During submission to GenBank, the final version of the assembly was annotated with the NCBI Prokaryotic Genome Annotation Pipeline (PGAP) v4.8 [22].

### 2.6. Genome Sequence Analysis

Genome coverage was analyzed by mapping all Illumina reads against the genome sequence, using the CLC Genomics Workbench v11 (Qiagen Aarhus A/S, Aarhus, Denmark). To confirm gene positions and contiguity, an assembly, using only Illumina reads, performed as explained above but excluding the option *--nanopore*, was compared by BLAST against the hybrid assembly, using UGENE v1.31 [23]. The accuracy, as determined by average nucleotide identities using BLAST algorithm (ANIb) [24], was calculated by pairwise comparisons between both genomes, using JSpeciesWS [25]. Additionally, variant calling analysis was done, with the CLC Genomics Workbench v11 (Minimum coverage 10, minimum count 2, and minimum frequency 75%), to detect discrepancies between Illumina reads and the final assembly. Genomic features associated with antibiotic resistances and virulence factors were detected and characterized, using the Pathosystems Resource Integration Center tool (PATRIC) v3.5.13 [26] and the software Resistance Gene Identifier (RGI) of the Comprehensive Antibiotic Resistance Database (CARD) [27]. Prediction of putative toxin-antitoxin systems (TAS) was done, using the prediction tool of the Type II Toxin-Antitoxin DataBase (TADB) [28].

### 2.7. Plasmid Sequence Characterization

Plasmid sequences were characterized, using three different approaches: the MOB-Typer tool from MOB-Suite software v1.4.9 [29], the web-based tool OriTfinder [30] and PlasmidFinder v2.0.2 [31]. The sequences were also analyzed by Nucleotide BLAST in NCBI.

### 2.8. Protein Expression Determination

*E. coli* strain CCUG 73778 was cultivated on Luria-Bertani (LB) broth, without added antibiotics, at 37 °C, overnight. Bacterial biomass was prepared for proteomics, by bead beating and subsequent digestion with trypsin, as described previously [32]. The LC-MS/MS analysis was performed as described previously [33]. The data were matched using Proteome Discoverer v1.4 (Thermo Fisher Scientific, Waltham, MA, USA), against the genome of *E. coli* strain CCUG 73778. Mascot 2.5 (Matrix Science, Boston, MA, USA) was used as a search engine with precursor mass tolerance of 5 ppm and fragment mass tolerance of 200 mmu and variable methionine oxidation. Fixed Value with a maximum delta Cn of 0.05 was employed in the database matching and the peptides used for protein identification were filtered at 1% false discovery rate (FDR). The analyses were performed in triplicate and lists of proteins identified by the peptides (specific for those proteins) were generated for each replicate.

### 2.9. Data Availability

The genome sequence data were deposited in GenBank under the accession numbers CP041337 to CP041343 (Supplementary Table S1).

## 3. Results and Discussion

### 3.1. Sequencing and Assembly Comparison

**Figure 1:**
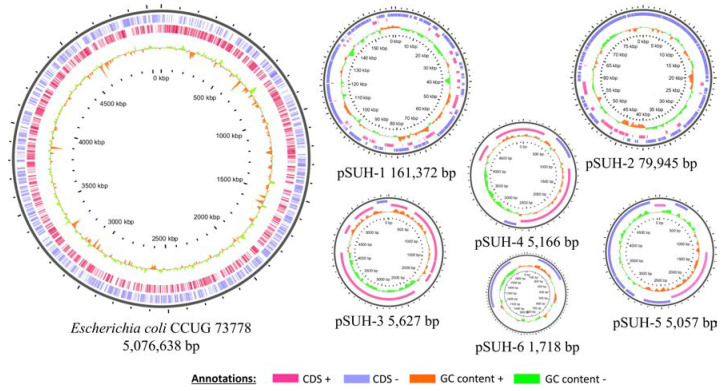
Genome architecture of ESBL-*E. coli* strain CCUG 73778 (one chromosome and six plasmids). From outside to inside, the circles represent the reverse coding sequences (CDSs) (blue), the direct CDSs (pink) and the GC content (green/orange).

*E. coli* strain CCUG 73778 was originally isolated from blood, although it was shown to be closely related to other isolates from the same outbreak which had been obtained from urine and feces samples of other infants [14]. The strain was identified as *E. coli* and characterized as the multi-locus sequence type (MLST)-serotype ST131-O25b, and observed to be carrying the CTX-M-15, TEM-1 and OXA-1-encoding genes [14]. Illumina sequencing yielded 3,320,002 paired-end reads (i.e., 1,660,001 pairs) with a read-length of 301 bp and a total of 999,320,602 bp (almost 200× coverage for a 5 Mb genome). After trimming, the Illumina sequences were assembled de novo, with SPAdes v3.11.1, generating a draft genome sequence, formed by 74 contigs with a total length of 5,279,300 bp and a GC-content of 50.8%. The length of the largest contig was observed to be 431,788 bp (Supplementary Figure S1A). On the other hand, the MinION sequencer produced 150,315 reads in 48 h, with an average length of 6625 bp and reads as large as 95,158 bp in length. The Phred quality score of these reads was 10.2 (i.e., probability of error of 0.095%). Albacore was able to base-call 97% of the raw reads in FAST5 to FASTQ format. These Oxford Nanopore reads were assembled, in combination with the Illumina reads, using SPAdes v3.11.1. The result was a draft genome sequence of seven contigs, with a total length of 5,335,523 bp and presenting the largest contig of 5 Mb (Supplementary Figure S1B). In this case, the GC content was 50.8%. From the seven contigs, two were not circularized. These two contigs, of 5 Mb and 79 kb, were closed with BLAST procedures, using the assembly generated only with Illumina reads as a database. The Oxford Nanopore sequencing yielded enough data to obtain a complete closed genome sequence formed by one contig of 5 Mb, which represented the chromosome, and the sequences of six plasmids, named pSUH 1–6 (plasmid Sahlgrenska University Hospital) (Figure 1). The annotation of the genome revealed 5393 genes, 5279 coding sequences (CDS), 214 pseudogenes, 7 complete ribosomal operons, 87 tRNAs and 5 ncRNAs.

Both assemblies were compared, by sequence identities, through ANIb values (Supplementary Table S2), to analyze the accuracy of the closed genome sequence. One of the main concerns about Oxford Nanopore sequence reads is the relatively high error rate that they exhibit, i.e., approximately 6% [34]; although, combining them with Illumina reads, which are characterized by having the lowest error rate of all NGS platforms [35], should compensate by correcting those errors during the assembly process. Some advantages of using Nanopore sequencing instead of other long-read sequencing platforms, such as PacBio, are that Nanopore sequencing does not require the large amounts of high-quality DNA, small plasmids can readily be detected if a non-size-selection-based library preparation kit is used, and the cost per sample is much less, particularly if using a barcoding kit for sequence determinations of multiple genomes. As can be seen in Supplementary Table S2, the hybrid assembly exhibited almost 100% sequence identity, with respect to the assembly using only Illumina reads. Additionally, no variants were detected between the Illumina reads and the final assembly. These results confirmed the accuracy and confidence of the hybrid assembly.

### 3.2. Antibiotic Resistance Factors

Using the software Resistance Gene Identifier (RGI) from the Comprehensive Antibiotic Resistance Database (CARD), 59 potential antibiotic resistance determinants were identified (Table 1). Only sequence matches classified as “perfect” or “strict” were considered for downstream descriptions, to decrease the possibility of false positives. The specific locus tags provided by PGAP annotation for each element are indicated in Supplementary Table S3. Among the 59 elements, 47 were localized on the chromosome, seven on the plasmid pSUH-1 and five on the pSUH-2.

Among the 47 chromosomally encoded elements are genes that encode for single elements or subunits of as many as 17 efflux pumps or transport proteins, indicating antibiotic transport out of the cell to be a major resistance mechanism among the identified chromosomal genes. Only ampC, a well-known chromosomally-encoded β-lactamase, possesses antibiotic inactivation as the mechanism of action. Additionally, *uhp*T and *glp*T are genes that encode two transporters involved in the uptake of phosphorylated sugars and represent a pathway through which fosfomycin can be internalized into the cell. Mutations on these elements can alter the affinity for fosfomycin and, hence, reduce its uptake into the cell. Mutations were found in UphT (E448K) and GlpT (E350Q), which have been described in the literature as being potentially related to an increase in the MIC of fosfomycin [36]. The rest of the genes identified in the chromosome by the RGI are regulators involved in the expression/repression of resistance-associated genes.

Among the plasmid-encoded resistance genes, three clinically-relevant β-lactamases were found: *bla*_TEM-1_ on pSUH-1; *bla*_OXA-1_ and the ESBL *bla*_CTX-M-15_, both localized on pSUH-2. As the β-lactamase genes are plasmid-encoded, they can be readily mobilized and transmitted between bacteria. Results of the whole, closed genome sequence are concordant with those of previous descriptions of *E. coli* strain CCUG 73778, wherein the presence of a plasmid of approximately 75 to 80 kb was observed by optical mapping, which was PCR-positive for *bla*_CTX-M-15_ [15].

Another gene to highlight is AAC(6′)-Ib-cr, which is important in the resistance against aminoglycosides and fluoroquinolones. The presence of this gene is often associated with the presence of specific mutations in the genes *gyrA* and *parC* [37], although no mutations were detected in these genes in *E. coli* strain CCUG 73778. The plasmid-encoded resistance genes, in both pSUH-1 and pSUH-2, have antibiotic inactivation as the most common mechanism of action (Table 1). Only one of the elements in the plasmids, *vgaC*, encoded in pSUH-1, is associated with antibiotic efflux activity. Considering all the resistance genes and mutations found in this genome, from the genomic point of view, the antibiotic resistance profile potentially confers resistance to a wide range of antibiotics to the bacterium, including aminoglycosides, β-lactams, macrolides, quinolones, tetracyclines, streptogramins, nitroimidazoles, diaminopyrimidines, sulfonamides, pleuromutilin and fosfomycin (Table 1).

### 3.3. Virulence Related Elements

The genome sequence of *E. coli* strain CCUG 73778 was analyzed, using the PATRIC database, to identify elements potentially related to virulence. Through this analysis, all putative ORFs encoded in the genome which could be related to known virulence determinants or virulence factors were highlighted. Virulence genes were detected only on the chromosome; no known virulence factors were detected on any of the plasmids. Several genes of *E. coli* strain CCUG 73778 were classified by PATRIC as being related to “adherence”, “invasion” and “motility”, as well as “biofilm formation” or “antiphagocytosis” (Table 2). Locus tags of the genes are indicated in Supplementary Table S4.

Among all elements found, four seem to have importance in urinary-tract infections (UTI). The first is the type I fimbriae operon (*fimABCDEFGHI*). Mutations in these genes have been shown to result in attenuated pathogenicity of uropathogenic *E. coli* strains (UPEC) [38]. The second element observed is represented by the gene clusters *papAIX* and *papCDFK*, related to the synthesis of pyelonephritis-associated pili (pap), a prevalent and important virulence factor among pyelonephritis-causing UPEC strains [39]. The third element is *wecE*, involved in the synthesis of the enterobacterial common antigen (ECA) [40]. Studies on *E. coli* have shown that *wecE* gene mutants were not able to produce the ECA polysaccharide, decreasing the colonization capacity of the strain in the urinary tract, suggesting a pathogenic-related function of ECA in UTI [38]. The last element observed is the arylsulfate sulfotransferase AssT, which is present in UPEC strains but absent in commensal strains [41,42], even though its role in virulence is still unclear.

Other genes were found that are more related to pathogenicity of other strain types, such as enterohaemorrhagic and enterotoxigenic *E. coli* strains (EHEC and ETEC). For example, two sets of genes commonly found in almost all strains of *E. coli*, the operon *ecpRABCDE* (*E. coli* Common Pilus) and the gene clusters *csgBAC* and *csgDEFG* (Curli formation). The first provides colonization and immune system evasion mechanisms that allow attachment and dissemination of EHEC strains within the host [43]. The *csg* gene clusters are expressed particularly among EHEC and ETEC strains [44] and have been associated with biofilm formation [45]. A homologue of the prepilin peptidase-dependent protein D (PpdD), the basic unit of the Type IV pilin (T4P), was also found in *E. coli* strain CCUG 73778. This factor can participate in pathogenic-associated processes, such as invasion of human epithelial cells, adhesion and promotion of biofilm formation in EHEC [46].

Finally, other elements that are highlighted include *eptC* (also called *yijP*) and *aslA* (arylsulfatase A), which have been shown to contribute to invasiveness of brain microvascular endothelial cells by *E. coli* K1 [47]; the gene clusters *cusCFBA* and *cusRS*, which relate to resistance against toxic levels of copper and silver [48,49,50]; *sitABCD*, associated in *E. coli* with resistance against H_2_O_2_ and necessary for complete virulence of *Salmonella typhimurium* [51]; *dsbAB*, specifically involved in the synthesis of virulence factors in different stages of infection [52]; and the gene *trxA*, which encodes Thioredoxin 1, a protein whose direct implication in virulence is not clear, but that has been shown to be involved in the stimulation of intracellular replication and virulence of *Salmonella enterica* [53]; and the capsule production genes, *kpsCDEFSTM*, considered to be an essential virulence determinant in several species of *Enterobacteriaceae* genera, such as *Klebsiella*, a causative agent of neonatal septicemia and meningitis [54,55,56].

### 3.4. Toxin–Antitoxin Systems

Toxin–antitoxin systems (TAS) are important elements that can be associated to the survival of cells in conditions of stress or for protection of mobile genetic elements [57,58,59,60] and, hence, can be important elements influencing pathogenicity. For instance, *Mycobacterium tuberculosis* strains can contain more than 70 TAS in their genomes [61], providing a clue that these systems may be highly important in pathogens. There are five kinds of TAS described nowadays, from which the type II is characterized by the fact that the toxin and the antitoxin are both translated into proteins [62].

In the genome of *E. coli* strain CCUG 73778, 28 putative type II TAS were found, using the TADB, 21 encoded in the chromosome, six in the pSUH-1 and one on the pSUH-2 (Supplementary Table S5). According to the toxin family assigned to the putative toxic element in each case (Table 3), the most abundant family observed was RelE, representing 25% of the total number of predicted TAS. The most common process targeted by these systems seems to be translation, thus affecting cell growth [63]. Two toxins belonging to the PemK family were found, one in pSUH-1 and one in pSUH-2. The toxin PemK has been related to plasmid maintenance, ensuring transmission of the plasmid during cell division, favoring the survival of only those cells that receive the plasmid [64]. In fact, it was the only TAS found in pSUH-2. Additionally, a toxin classified in the MosT family was found. The toxin MosT is described as an element that could be related to the maintenance of integrative conjugative elements (ICE), such as the SXT, originally described in the pathogen *Vibrio cholerae* [65]. On the other hand, one of the putative ORFs could not be assigned to any known family and two putative TAS were difficult to classify, since both elements of each system were carrying domains related to antitoxins (Table 3).

### 3.5. Plasmid Sequence Characterization

Analyses of the plasmid sequences using MOB-typer, OriTfinder and Plasmid Finder were done to obtain comprehensive characterizations of the plasmids, including identification of the replicon type (Inc family), relaxase type (MOB type) and mate pair formation type (MPF, which depends on the type IV secretion system (T4SS) sequence), as well as detection of origin of transfer (OriT) and prediction the potential mobility (Table 4). The plasmids pSUH-1 and pSUH-2 belong to IncF family and to MOB_F_ and MPF_F_ type. MOB_F_ relaxases are commonly found in large plasmids and the IncF plasmids are typically responsible for transmission of antibiotic resistance [66,67], results that are in concordance with the characteristics of the mentioned plasmids. Plasmids pSUH-3, pSUH-4 and pSUH-5 were classified as ColRNAI replicon cluster 1857 (for pSUH-3 and 5) and cluster 1291 (for pSUH-4). MOB_Q_ and MOB_P_ types were detected, which are present in plasmids of different sizes and commonly found in small mobilizable plasmids [67,68]. Additionally, all plasmids, except pSUH-4, have OriT.

Plasmids pSUH-1 and pSUH-2 have been categorized by MOB-typer as self-transmissible (conjugative), as they encode all the required elements, a relaxase, a T4SS, a type IV coupling protein (T4CP) and an OriT [69] (Table 4). A plasmid is considered “mobilizable” if it has the OriT and/or a relaxase. The OriT is one of the most important factors for mobilization, as other necessary proteins, like the relaxase, can be provided in trans by genes encoded in other plasmids present in the same bacteria [69,70,71]. According to this information, plasmids pSUH-3, 5 and 6 were classified as mobilizable. The plasmid pSUH-4 was also classified as mobilizable, even though no OriT was found by either of two approaches (MOB-typer and OriTfinder). This is because MOB-typer determines that a plasmid is mobilizable if it has a relaxase or an OriT.

BLAST analysis of the plasmid sequences in GenBank revealed the presence of similar sequences in other genomes. Firstly, pSUH-1 exhibited four matches, covering more than 95% of the sequence with identities greater than 99.9%, with plasmids present in clinical *E. coli* isolates from Sweden (CP023845.1), South Korea (CP024831.1 and CP024816.1) and the USA (CP025708.1). Secondly, pSUH-2 exhibited two matches, covering more than 96% of the sequence, with a plasmid harbored in the multidrug resistant strain *Klebsiella pneumoniae* strain KP_112126 [72] and with plasmids harbored in *bla*_CTX-M-15_ producing *E. coli* strains isolated in Canada [73], with identities greater than 99%, in both cases. The most remarkable part of pSUH-2 is a sequence between positions 43,211 and 59,126, containing several regions flanked by transposases. This sequence includes the ESBL gene *bla*_CTX-M-15_ and the β-lactamase *bla*_OXA-1__,_ among other putative antibiotic resistance factors.

In the cases of pSUH-3, pSUH-4 and pSUH-6, almost identical plasmids in size and sequence can be found in other *E. coli* strains (99% identity in 99% of the sequence) isolated around the world. As an example, pSUH-3 is highly similar to plasmids in *E. coli* isolates from Australia (CP035482.1), USA (CP006787.1), Norway (LM997244.1), France (LT985311.1) and Brazil (CP035378.1). Plasmids almost identical to pSUH-4 can be found, as well, in isolates described in Italy (MG649065.1), USA (CP006786.1) and Sweden (JX238459.1); several of the isolates represent UPEC, carbapenemase- and ESBL-producing strains. As mentioned above, pSUH-4 was not harboring an OriT, but still appears in isolates from different countries and with highly similar matches with plasmids of *Klebsiella pneumoniae*, which could mean that the plasmid contains a yet not described OriT. The most representative match for pSUH-6 is the plasmid pTB511 (CP034830.1), which has the same size (1718 bp) and is present in the colistin-resistant strain *Salmonella* sp. SSDFZ54 (ASM401073v1), isolated in China.

The plasmid pSUH-5 exhibits identities greater than 90%, over 60–65% of its sequence length, with plasmids belonging to strains of *E. coli* and *Salmonella* spp. Among all BLAST results generated, a gap of approximately 1700 bp was observed, from position 1100 to 2800 of the pSUH-5 sequence that did not generate any similarity matches at first sight. This region was extracted from the sequence and analyzed alone by BLAST. Only three significant results were obtained, exhibiting similarities ranging from 80% to 90% over almost 100% of the fragment length. These matches belonged to the plasmid pSZECL (CP035127.1) of the carbapenemase producer *Enterobacter cloacae* strain SZECL1 (91% identity), plasmid pEC25-4 of the carbapenem-resistant *E. coli* strain EC25 (84% identity), and plasmid p5 (CP033951.1) from the *Klebsiella pneumoniae* subsp. *pneumoniae* strain ARLG-3135 (84% identity). This sequence region encodes a mobilization protein A and two hypothetical proteins. Deeper analysis did not find any known functional domains on these two hypothetical proteins, although the same protein sequences have been found encoded in other *E. coli*, *K. pneumoniae*, *Shigella fleixneri* and *Salmonella enterica* strains.

### 3.6. Protein Expression

Even though the genomic description of all observed features is of great help to understand what a specific strain could do, potentially, analyses at the protein level are also important to determine what is actually expressed, establishing the important link between genome sequence and protein expression and function. One of the main interests of this study was the expression of genes under no stress on the bacterium.

Triplicate MS analyses of biomass from cultures in late stationary phase of *E. coli* strain CCUG 73778 were performed, in order to determine if the elements described in the genomic analysis, which are presumably involved in resistance, virulence and TAS, were expressed in vivo. Many proteins were observed to be expressed without exposure to antibiotics or stress produced by host-associated response mechanisms (Table S6), which suggests that these features are expressed constitutively, even though expression levels were low, in some cases.

Regarding antibiotic resistance, as many as 20 chromosomally-encoded resistance-associated factors, mostly efflux pumps and membrane associated proteins, were observed to be expressed, without the presence of antibiotics. Furthermore, expression of all relevant resistance genes against macrolides, aminoglycosides and β-lactams, which are plasmid-encoded, were detected, including the genes encoding the β-lactamases, *bla*_OXA-1_, *bla*_TEM-1_ and the ESBL *bla*_CTX-M15_.

Additionally, the expressions of as many as 10 virulence-associated elements and 14 components of the TAS were also confirmed. In the case of TAS, toxins were mostly detected, with special mention to DUF1778 (FMA85_20310) and DUF2559 (FMA85_20770), which are proteins with unknown function. The importance of proteins with domains with unknown function has been increasing in recent years, as a huge percentage of proteins in bacteria have domains with functions not yet characterized, some of them shared, even among different kingdoms [74]. In this study, these two proteins were not only characterized from the sequence point of view, but their expression at protein level was also demonstrated. As they are expressed constitutively, they might have an essential function in the cell metabolism. According to the TAS analysis, these two proteins could represent the antitoxin component of their respective putative systems, although further studies will be necessary to determine the real role of these proteins.

## 4. Conclusions

Combining Illumina (short-read) and Oxford Nanopore (long-read) sequences in a hybrid assembly allows comprehensive construction of high-quality genome sequences, negating the inherent error rate of Oxford Nanopore sequence reads, as expected. In the case of this study, a high-quality genome sequence of the nosocomial, virulent, ESBL-producing *E. coli* strain CCUG 73778 was generated, allowing study of the structure of the entire genome, based on one chromosome and six plasmids, as well as the genomic features. With respect to the antibiotic multi-resistance of the strain, the genome encodes 59 putative resistance determinants for a wide range of antibiotics. It also encodes an extensive range of virulence-associated elements, as well as 28 TAS, that could participate in benefiting capacity for developing infection and propagation. Plasmid sequences were characterized comprehensively and determined to be conjugative or mobilizable, reflecting capacities for transfer to other bacteria, potentially contributing to the widespread dissemination of antibiotic resistance genes. Additionally, plasmid sequences from isolates from around the world, with high similarities to the plasmids of *E. coli* strain CCUG 73778, were found in the nucleic acid sequence databases, exemplifying the global problem of transmission of resistance genes through mobility of plasmids. Expression with no induction of many of the described genomic elements regarding antibiotic resistance, virulence and toxin-antitoxin systems was confirmed by proteomic analyses. This complete, closed genome represents an important reference, providing detailed genetic information and proteomic data that will be used as a model for characterizing antibiotic resistance mechanisms and interactions of different elements, as well as for the study and control of the spread of bacteria carrying plasmids harboring ESBL genes in the hospital environment. Further studies are ongoing to confirm the specific functionality and expression under different conditions, including exposure to antibiotics, highlighting differences in levels of protein expression and up- or down-regulation of particular genes or even pathways encoding hypothetical proteins, thus illuminating the relevance of the genomic features described in this work.

## Figures and Tables

**Table 1:** Resistance determinants detected in each replicon exhibiting antibiotic resistance genes. Each gene is associated with the drug for which it encodes resistance and the mechanism of action used in each case.

Replicon	System/Gene	Drug Class	Resistance Mechanism
Chromosome	*acrD*	aminoglycoside antibiotic	antibiotic efflux
	*mdtNOP*	acridine dye; nucleoside antibiotic	antibiotic efflux
	*mdtABC-tolC*	aminocoumarin antibiotic	antibiotic efflux
	*mdtG*	fosfomycin	antibiotic efflux
	*mdtEF-tolC*	multidrug	antibiotic efflux
	*mdtH*	fluoroquinolone antibiotic	antibiotic efflux
	*patA*	fluoroquinolone antibiotic	antibiotic efflux
	*emrAB-tolC*	fluoroquinolone antibiotic	antibiotic efflux
	*emrE*	macrolide antibiotic	antibiotic efflux
	*acrAB-tolC*	multidrug	antibiotic efflux
	*acrEF-tolC*	multidrug	antibiotic efflux
	*emrKY-tolC*	tetracycline antibiotic	antibiotic efflux
	*mdfA*	multidrug	antibiotic efflux
	*msbA*	nitroimidazole antibiotic	antibiotic efflux
	*msrB*	macrolide antibiotic; streptogramin antibiotic	antibiotic efflux
	*bacA*	nitroimidazole antibiotic	antibiotic efflux
	*yojL*	peptide antibiotic	antibiotic efflux
	*glpT* (mutation E350Q)	fosfomycin	antibiotic target alteration
	*uhpT* (mutation E448K)	fosfomycin	antibiotic target alteration
	*pmrEFC* and *arnA* (*pmrL*)	peptide antibiotic	antibiotic target alteration
	*ampC*	cephalosporin; penam	antibiotic inactivation
	*kdpE*, *cpxA*, *baeRS*, *soxRS*, *gadX*, *gadW*, *evgSA*, *CRP*, *H-HS*, *mdtE*, *emrR*, *acrS*, *ptsI*	///	regulators
pSUH1	*aadA5*	aminoglycoside antibiotic	antibiotic inactivation
	*bla*_TEM-1_	cephalosporin; monobactam; penam; penem	antibiotic inactivation
	*dfrA17*	diaminopyrimidine antibiotic	antibiotic target replacement
	*mphA, Mrx*	macrolide antibiotic	antibiotic inactivation
	*vgaC*	streptogramin antibiotic; pleuromutilin antibiotic	antibiotic efflux
	*sul1*	sulfonamide antibiotic	antibiotic target replacement
pSUH2	AAC(3)-IIc	aminoglycoside antibiotic	antibiotic inactivation
	*bla*_CTX-M-15_	cephalosporin	antibiotic inactivation
	*bla*_OXA-1_	cephalosporin; penam	antibiotic inactivation
	AAC(6′)-Ib-cr	fluoroquinolone antibiotic; aminoglycoside antibiotic	antibiotic inactivation
	*catB3*	phenicol antibiotic	antibiotic inactivation

**Table 2:** Virulence associated elements detected in the chromosome. Each gene or group of genes is associated with the function assigned by Pathosystems Resource Integration Center (PATRIC) database.

Contig	Genes Detected	Function (PATRIC)
Chromosome	*ecpRABCDE*,	Adherence
	*csgBAC* and *csgDEFG*	Adherence
	*fimABCDEFGHI*	Adherence, invasion, virulence
	*papAIX, papCDFK*	Adherence
	*ppdD*	Invasion, adhesion, biofilm, cell motility
	*eptC*	Invasion
	*aslA*	Invasion
	*cusCFBA* and *cusRS*	Invasion
	*kpsCDEFSTM*	Invasion, antiphagocytosis
	*dsbAB*	Virulence
	*sit* *ABCD*	Virulence
	*ompF*	Virulence
	*wecE*	Virulence
	*assT*	Virulence
	*trxA*	Modulate host immune response

**Table 3:** Toxin–antitoxin systems (TAS) found in the genome sequence of *E. coli* strain CCUG 73778, classified by toxin family. The number of representatives of each family, as well as the cellular process that is targeted in each case, are indicated.

Toxin Family	Number	Cellular Process
COG2929/SplT	1	translation
Fic	3	translation
GNAT	1	translation
HipA	2	translation
MazF	3	translation
PIN	2	translation
RelE	7	translation
YeeV	3	cytoskeleton
MosT	1	protection of ICE
PemK	2	plasmid maintenance
unclear	2	ND *
null	1	ND *

* ND = Not determined.

**Table 4:** Characterizations of the six plasmid sequences harbored in the *E. coli* strain CCUG 73778, according to replicon type, relaxase type, Mate-Pair Formation (MPF) type, presence of OriT and predicted mobility.

Plasmid	Size (bp)	Replicon Types	Relaxase Type	MPF Type	OriT	Predicted Mobility
pSUH-1	161,372	IncFIA, IncFIB	MOB_F_	MPF_F_	Yes	Conjugative
pSUH-2	79,945	IncFIIA, IncFII	MOB_F_	MPF_F_	Yes	Conjugative
pSUH-3	5627	ColRNAI rep cluster 1857	MOB_P_	-	Yes	Mobilizable
pSUH-4	5166	ColRNAI rep cluster 1291	MOB_Q_	-	ND *	Mobilizable
pSUH-5	5057	ColRNAI rep cluster 1857	MOB_P_	-	Yes	Mobilizable
pSUH-6	1718	-	-	-	Yes	Mobilizable

* ND = Not Detected.

## Supplementary Materials

The following are available online at https://www.mdpi.com/2076-2607/8/6/893/s1, Table S1. GenBank accession numbers of the different replicons found in *E. coli* strain CCUG 73778; Table S2. ANIb comparison between the genome sequences obtained in the Illumina and hybrid assemblies. Table S3. Locus tags and annotations of the different putative antibiotic resistance-associated elements identified by RGI, as well as the replicon where they are encoded. Table S4. Locus tags and annotations of the different virulence-associated elements identified by PATRIC. Table S5. Locus tags and annotations of all detected putative toxin-antitoxin systems (TAS). Table S6. Number of specific peptides detected by proteomics analyses for genomic elements in each replicate (A, B, C). Figure S1. Plots showing the cumulative lengths (*y*-axis) of genome assembly contigs, ordered from the largest to the smallest (*x*-axis).
