# Supplementary material for: Genomic and Proteomic Characterization of the Extended-Spectrum β-Lactamase (ESBL)-Producing Escherichia coli Strain CCUG 73778: A Virulent, Nosocomial Outbreak Strain
Source: Microorganisms. 2020 Jun 13;8(6):893. doi: 10.3390/microorganisms8060893 (PMC7355845; doi:10.3390/microorganisms8060893)
Supplement: Supplementary file 1 [file microorganisms-08-00893-s001.pdf]

Table S1. GenBank accession numbers of the different replicons found in *E. coli* strain CCUG 73778.

Replicon	Accesion number
Chromosome	CP041337
Plasmid pSUH-1	CP041338
Plasmid pSUH-2	CP041339
Plasmid pSUH-3	CP041340
Plasmid pSUH-4	CP041341
Plasmid pSUH-5	CP041342
Plasmid pSUH-6	CP041343

Table S2. ANIb comparison between the genome sequences obtained in the Illumina and hybrid assemblies. Results show a high level of identity between the two sequences. The first number indicates the percentages of similarity of the aligned sequence regions. The number in brackets indicates the percentage of sequences aligned.

.	ANIb values (%) and coverage of aligned sequence [%]	
	**Illumina assembly**	**Hybrid assembly**
**Illumina assembly**	*	100.00 [99.7%]
**Hybrid assembly**	99.99 [99.1%]	*

Table S3. Locus tags and annotations of the different putative antibiotic resistance-associated elements identified by RGI, as well as the replicon where they are encoded. The elements that correspond to mutations are indicated

Replicon	Best RGI Hit	Locus tag	Product annotation
Chromosome	*mdtN*	FMA85_16810	multidrug efflux transporter periplasmic adaptorsubunit MdtN
	mdtO	FMA85_16815	multidrug efflux transporter permease subunit MdtO
	mdtP	FMA85_16820	multidrug efflux transporter outer membrane subunit MdtP
	mdtC	FMA85_02700	multidrug efflux RND transporter permease subunit MdtC
	mdtB	FMA85_02705	multidrug efflux RND transporter permease subunit MdtB
	mdtA	FMA85_02710	multidrug efflux RND transporter subunit MdtA
	cpxA	FMA85_17820	envelope stress sensor histidine kinase CpxA
	baeR	FMA85_02685	two-component system response regulator BaeR
	baeS	FMA85_02690	two-component system sensor histidine kinase BaeS
	acrD	FMA85_00555	multidrug efflux RND transporter permease AcrD
	kdpE	FMA85_11255	two-component system response regulator KdpE
	ampC β-lactamase	FMA85_16410	BlaEC family class C beta-lactamase
	mdtH	FMA85_08710	multidrug efflux MFS transporter MdtH
	pata	FMA85_22175	putrescine aminotransferase
	emrB	FMA85_24945	multidrug efflux MFS transporter permease subunit EmrB
	emrA	FMA85_24950	multidrug efflux MFS transporter periplasmic adaptor subunit EmrA
	emrR	FMA85_24955	multidrug efflux transporter EmrAB transcriptional repressor EmrR
	ptsI (mutation)	FMA85_00770	phosphoenolpyruvate-protein phosphotransferase PtsI
	mdtG	FMA85_08770	multidrug efflux MFS transporter MdtG
	glpT (mutation)	FMA85_01615	glycerol-3-phosphate transporter
	uhpT (mutation)	FMA85_19130	hexose-6-phosphate:phosphate antiporter
	emrE	FMA85_03765	SMR family multidrug efflux protein EmrE
	msrB	FMA85_04850	peptide-methionine (R)-S-oxide reductase MsrB
	marA	FMA85_05935	MDR efflux pump AcrAB transcriptional activator MarA
	marR (mutation)	FMA85_05940	multiple antibiotic resistance transcriptional regulator MarR
	H-NS	FMA85_07425	DNA-binding transcriptional regulator H-NS
	evgS	FMA85_01005	acid-sensing system histidine kinase EvgS
	evgA	FMA85_01010	acid-sensing system DNA-binding response regulator EvgA
	mdfA	FMA85_10505	multidrug efflux MFS transporter MdfA
	acrR (mutation)	FMA85_12355	multidrug efflux transporter transcriptional repressor AcrR
	acrA	FMA85_12360	multidrug efflux RND transporter periplasmic adaptor subunit AcrA
	acrB	FMA85_12365	multidrug efflux RND transporter permease subunit
	mdtM	FMA85_15105	multidrug efflux MFS transporter MdtM
	soxR (mutation)	FMA85_16945	redox-sensitive transcriptional activator SoxR
	soxS (mutation)	FMA85_16950	superoxide response transcriptional regulator SoxS
	gadX	FMA85_19970	acid resistance transcriptional activator GadX
	gadW	FMA85_19975	acid resistance transcriptional activator GadW
	mdtF	FMA85_19980	multidrug efflux RND transporter permease subunit
	mdtE	FMA85_19985	multidrug transporter subunit MdtE
	CRP	FMA85_20795	cAMP-activated global transcriptional regulator CRP
	acrF	FMA85_21255	multidrug efflux RND transporter permease subunit AcrF
	acrE	FMA85_21260	multidrug efflux RND transporter periplasmic adaptor subunit AcrE

Continuation of Table S3. Locus tags and annotations of the different putative antibiotic resistance-associated elements identified by RGI, as well as the replicon where they are encoded. The elements that correspond to mutations are indicated

Replicon	Best Hit	Locus tag	Product annotation
Chromosome	*acrS*	FMA85_21265	multidrug efflux transporter transcriptional repressor AcrS
	tolC	FMA85_22375	outer membrane channel protein TolC
	msbA	FMA85_09370	lipid A ABC transporter ATP-binding protein/permease MsbA
	arnA	FMA85_01535	bifunctional UDP-4-amino-4-deoxy-L-arabinose formyltransferase/UDP-glucuronic acid oxidase ArnA
	pmrF	FMA85_01540	undecaprenyl-phosphate 4-deoxy-4-formamido-L-arabinose transferase
	pmrC	FMA85_16645	phosphoethanolamine transferase EptA
	yojI	FMA85_01750	microcin J25 efflux ABC transporter YojI
	bacA	FMA85_22260	undecaprenyl-diphosphate phosphatase
	pmrE	FMA85_02940	UDP-glucose 6-dehydrogenase
	emrK	FMA85_01015	multidrug efflux MFS transporter periplasmic adaptor subunit EmrK
	emrY	FMA85_01020	multidrug efflux MFS transporter permease subunit EmrY
pSUH-1	*aadA5*	FMA85_25675	ANT(3")-Ia family aminoglycoside nucleotidyltransferase AadA5
	$bla_{\text{TEM-1}}$	FMA85_25740	class A broad-spectrum beta-lactamase TEM-1
	dfrA17	FMA85_25680	trimethoprim-resistant dihydrofolate reductase DfrA17
	mphA	FMA85_25610	Mph(A) family macrolide 2'-phosphotransferase
	mrx	FMA85_25615	MFS transporter
	vgaC	FMA85_25305	AbrB/MazE/SpoVT family DNA-binding domain-containing protein
	sul1	FMA85_25665	sulfonamide-resistant dihydropteroate synthase Sul1
pSUH-2	AAC(3)-IIc	FMA85_26690	aminoglycoside N-acetyltransferase AAC(3)-IIa
	$bla_{\text{CTX-M-15}}$	FMA85_26660	class A extended-spectrum beta-lactamase CTX-M-15
	$bla_{\text{OXA-1}}$	FMA85_26705	oxacillin-hydrolyzing class D beta-lactamase OXA-1
	AAC(6')-Ib-cr	FMA85_26710	fluoroquinolone-acetylating aminoglycoside 6'-N-acetyltransferase AAC(6')-Ib-cr5
	catB3	FMA85_26700	CatB-related O-acetyltransferase

Table S4. Locus tags and annotations of the different virulence-associated elements identified by PATRIC. All elements were chromosomally-encoded

Gene	Locus tag	Product annotation
ecpR	FMA85_13180	EcpR
ecpA	FMA85_13185	EcpA
ecpB	FMA85_13190	EcpB
ecpC	FMA85_13195	hypothetical protein (EcpC)
ecpD	FMA85_13200	EcpD
ecpE	FMA85_13205	EcpE
cusA	FMA85_11940	CusA
cusB	FMA85_11945	CusB
cusF	FMA85_11950	CusF
cusC	FMA85_11955	CusC
cusR	FMA85_11960	CusR
cusS	FMA85_11965	CusS
ppdD	FMA85_14220	PpdD
fimB	FMA85_15240	FimB
fimE	FMA85_15235	FimE
fimA	FMA85_15230	FimA
fimI	FMA85_15225	type 1 fimbrial protein (FimI)
fimC	FMA85_15220	FimC
fimD	FMA85_15215	fimbrial biogenesis outer membrane usher protein (FimD)
fimF	FMA85_15210	FimF
fimG	FMA85_15205	FimG
fimH	FMA85_15200	FimH
papC	FMA85_01145	fimbrial biogenesis outer membrane usher protein
papD	FMA85_01150	fimbria/pilus periplasmic chaperone
papF	FMA85_01155	hypothetical protein
papK	FMA85_01165	fimbrial protein
papI	FMA85_23325	pilus assembly protein
papX	FMA85_23345	MarR family transcriptional regulator
papA	FMA85_23335	type 1 fimbrial protein
wecE	FMA85_18525	RffA (WecE)
dctA	FMA85_19910	dicarboxylate/amino acid:cation symporter
dsbA	FMA85_18085	DsbA
dsbB	FMA85_07890	DsbB
sitA	FMA85_08055	SitA
sitB	FMA85_08060	SitB
sitC	FMA85_08065	SitC
sitD	FMA85_08070	SitD
ompF	FMA85_09295	OmpF
assT	FMA85_22360	aryl-sulfate sulfotransferase
trxA	FMA85_18575	thioredoxin TrxA

Continuation Table S4. Locus tags and annotations of the different virulence-associated elements identified by PATRIC. All elements were chromosomally-encoded

Gene	Locus tag	Product annotation
csgB	FMA85_08825	CsgB
csgA	FMA85_08820	CsgA
csgC	FMA85_08815	CsgC
csgD	FMA85_08830	CsgD
csgE	FMA85_08835	CsgE
csgF	FMA85_08840	CsgF
csgG	FMA85_08845	CsgG
eptC	FMA85_17600	phosphoethanolamine transferase CptA
aslA	FMA85_18470	arylsulfatase
kpsC	FMA85_22885	capsular polysaccharide biosynthesis protein
kpsD	FMA85_22895	polysialic acid transporter KpsD
kpsE	FMA85_22900	capsule polysaccharide export inner-membrane protein KpsE
kpsF	FMA85_22905	KpsF/GutQ family sugar-phosphate isomerase
kpsS	FMA85_22880	capsular biosynthesis protein
kpsT	FMA85_22845	ABC transporter ATP-binding protein
kpsM	FMA85_22840	ABC transporter permease

Table S5. Locus tags and annotations of all detected putative toxin-antitoxin systems (TAS). The replicon which encodes each system is also indicated.

Replicon	TAS nº	Toxin family	Locustag	Product annotation
Chromosome	TAS 1	RelE	FMA85_03005	Txe/YoeB family addiction module toxin
			FMA85_03000	YoeB-YefM toxin-antitoxin system antitoxin YefM
	TAS 2	YeeV	FMA85_03095	type IV toxin-antitoxin system YeeU family antitoxin
			FMA85_03090	type IV toxin-antitoxin system toxin CbtA
	TAS 3	HipA	FMA85_06005	type II toxin-antitoxin system serine/threonine protein kinase toxin HipA
			FMA85_06000	type II toxin-antitoxin system antitoxin HipB
	TAS 4	unclear	FMA85_09475	helix-turn-helix domain-containing protein
			FMA85_09480	DNA-binding protein
	TAS 5	PIN	FMA85_09940	tyrosine-type recombinase/integrase
			FMA85_09935	helix-turn-helix transcriptional regulator
	TAS 6	RelE	FMA85_12250	type II toxin-antitoxin system RelE/ParE family toxin
			FMA85_12245	HigA family addiction module antidote protein
	TAS 7	mosT	FMA85_13240	nucleotidyl transferase AbiEii/AbiGii toxin family protein
			FMA85_13245	hypothetical protein
	TAS 8	unclear	FMA85_13650	endonuclease/exonuclease/phosphatase family protein
			FMA85_13645	class I SAM-dependent methyltransferase
	TAS 9	MazF	FMA85_14525	plasmid maintenance protein CcdB
			FMA85_14530	antitoxin
	TAS 10	COG2929/SplT	FMA85_15055	BrnT family toxin
			FMA85_15050	BrnA antitoxin family protein
	TAS 11	YeeV	FMA85_15450	type IV toxin-antitoxin system YeeU family antitoxin
			FMA85_15445	toxin
	TAS 12	MazF	FMA85_15995	endoribonuclease toxin ChpB
			FMA85_16000	type II toxin-antitoxin system ChpS family antitoxin
	TAS 13	HipA	FMA85_16130	toxin HipA
			FMA85_16135	helix-turn-helix domain-containing protein
	TAS 14	RelE	FMA85_17955	type II toxin-antitoxin system RelE/ParE family toxin
			FMA85_17950	helix-turn-helix domain-containing protein
	TAS 15	Null	FMA85_18695	DHA2 family efflux MFS transporter permease subunit
			FMA85_18690	FadR family transcriptional regulator
	TAS 16	RelE	FMA85_19155	type II toxin-antitoxin system RelE/ParE family toxin
			FMA85_19150	DNA-binding transcriptional regulator
	TAS 17	GNAT	FMA85_20315	GNAT family N-acetyltransferase
			FMA85_20310	DUF1778 domain-containing protein
	TAS 18	Fic	FMA85_20775	putative adenosine monophosphate-protein transferase Fic
			FMA85_20770	DUF2559 family protein
	TAS 19	RelE	FMA85_21920	type II toxin-antitoxin system YhaV family toxin
			FMA85_21925	type II toxin-antitoxin system PrlF family antitoxin
	TAS 20	RelE	FMA85_22125	mRNA interferase HigB
			FMA85_22130	type II toxin-antitoxin system antitoxin HigA
	TAS 21	YeeV	FMA85_22940	type IV toxin-antitoxin system YeeU family antitoxin
			FMA85_22935	toxin

Continuation Table S5. Locus tags and annotations of all detected putative toxin-antitoxin systems (TAS). The replicon which encodes each system is also indicated

Replicon	TAS nº	Toxin family	Locustag	Product annotation
pSUH-1	TAS 22	MazF	FMA85_25270	type II toxin-antitoxin system toxin CcdB
			FMA85_25275	type II toxin-antitoxin system antitoxin CcdA
	TAS 23	PIN	FMA85_25310	type II toxin-antitoxin system VapC family toxin
			FMA85_25305	AbrB/MazE/SpoVT family DNA-binding domain-containing protein
	TAS 24	Fic	FMA85_25355	type II toxin-antitoxin system death-on-curing family toxin
			FMA85_25350	type II toxin-antitoxin system Phd/YefM family antitoxin
	TAS 25	Fic	FMA85_25355	type II toxin-antitoxin system death-on-curing family toxin
			FMA85_25360	PdcA protein
	TAS 26	RelE	FMA85_25460	type II toxin-antitoxin system RelE/ParE family toxin
			FMA85_25465	ribbon-helix-helix protein, CopG family
	TAS 27	PemK	FMA85_25765	mRNA interferase PemK
			FMA85_25770	antitoxin PemI
pSUH-2	TAS 28	PemK	FMA85_26725	mRNA interferase PemK
			FMA85_26730	antitoxin PemI

Table S6. Number of specific peptides detected by proteomics analyses for genomic elements in each replicate (A, B, C). The peptides unique for particular proteins are used for identification of that protein. Locus tag and replicon of origin are indicated. Only elements for which peptides have been detected in, at least, two replicates are indicated.

Category	Replicon	NCBI locus stag	Description	A	B	C
Antibiotic resistances	Chromosome	FMA85_17820	envelope stress sensor histidine kinase CpxA	3	4	2
	Chromosome	FMA85_02685	two-component system response regulator BaeR	4	5	3
	Chromosome	FMA85_02690	two-component system sensor histidine kinase BaeS		2	2
	Chromosome	FMA85_11255	two-component system response regulator KdpE	1	1	
	Chromosome	FMA85_24950	multidrug efflux MFS transporter periplasmic adaptor subunit EmrA	7	8	5
	Chromosome	FMA85_24955	multidrug efflux transporter EmrAB transcriptional repressor EmrR	3	2	4
	Chromosome	FMA85_00770	phosphoenolpyruvate-protein phosphotransferase PtsI	29	31	33
	Chromosome	FMA85_01615	glycerol-3-phosphate transporter	1	1	2
	Chromosome	FMA85_04850	peptide-methionine (R)-S-oxide reductase MsrB	2	2	1
	Chromosome	FMA85_07425	DNA-binding transcriptional regulator H-NS	9	9	9
	Chromosome	FMA85_01010	acid-sensing system DNA-binding response regulator EvgA	1		2
	Chromosome	FMA85_12360	multidrug efflux RND transporter periplasmic adaptor subunit AcrA	14	13	15
	Chromosome	FMA85_12365	multidrug efflux RND transporter permease subunit	14	14	13
	Chromosome	FMA85_19980	multidrug efflux RND transporter permease subunit	8	9	14
	Chromosome	FMA85_19985	multidrug transporter subunit MdtE	13	12	13
	Chromosome	FMA85_20795	cAMP-activated global transcriptional regulator CRP	10	10	12
	Chromosome	FMA85_22375	outer membrane channel protein TolC	19	19	19
	Chromosome	FMA85_09370	lipid A ABC transporter ATP-binding protein/permease MsbA	6	7	6
	Chromosome	FMA85_01535	bifunctional UDP-4-amino-4-deoxy-L-arabinose formyltransferase/UDP-glucuronic acid oxidase ArnA	12	11	11
	Chromosome	FMA85_01540	undecaprenyl-phosphate 4-deoxy-4-formamido-L-arabinose transferase	1	1	1
	pSUH-1	FMA85_25740	class A broad-spectrum beta-lactamase TEM-1	18	17	18
	pSUH-1	FMA85_25680	trimethoprim-resistant dihydrofolate reductase DfrA17	3	2	3

Continuation Table S6. Number of specific peptides detected by proteomics analyses for genomic elements in each replicate (A, B, C). The peptides unique for particular proteins are used for identification of that protein. Locus tag and replicon of origin are indicated. Only elements for which peptides have been detected in, at least, two replicates are indicated.

Category	Replicon	NCBI locus tag	Description	A	B	C
**Antibiotic resistance**	pSUH-1	FMA85_25610	Mph(A) family macrolide 2'-phosphotransferase	6	8	8
	pSUH-2	FMA85_26690	aminoglycoside N-acetyltransferase AAC(3)-IIa	5	6	5
	pSUH-2	FMA85_26660	class A extended-spectrum beta-lactamase CTX-M-15	16	17	18
	pSUH-2	FMA85_26705	oxacillin-hydrolyzing class D beta-lactamase OXA-1	8	6	7
	pSUH-2	FMA85_26710	fluoroquinolone-acetylating aminoglycoside 6'-N-acetyltransferase AAC(6')-Ib-cr5	6	6	6
**Virulence**	Chromosome	FMA85_11965	cusS		1	1
	Chromosome	FMA85_19910	dicarboxylate/amino acid:cation symporter	1	2	1
	Chromosome	FMA85_08055	sitA		1	1
	Chromosome	FMA85_09295	ompF	2	1	3
	Chromosome	FMA85_18575	thioredoxin TrxA	23	24	22
	Chromosome	FMA85_17600	phosphoethanolamine transferase CptA	26	27	26
	Chromosome	FMA85_22885	capsular polysaccharide biosynthesis protein	1	1	1
	Chromosome	FMA85_22895	polysialic acid transporter KpsD	14	11	15
	Chromosome	FMA85_22900	capsule polysaccharide export inner-membrane protein KpsE	5	8	4
	Chromosome	FMA85_22905	KpsF/GutQ family sugar-phosphate isomerase		1	1
**TA systems**	Chromosome	FMA85_13650	endonuclease/exonuclease/phosphatase family protein	1	1	2
	Chromosome	FMA85_15995	endoribonuclease toxin ChpB	8	8	8
	Chromosome	FMA85_18695	DHA2 family efflux MFS transporter permease subunit	2	3	4
	Chromosome	FMA85_20310	DUF1778 domain-containing protein	2	2	1
	Chromosome	FMA85_20775	putative adenosine monophosphate-protein transferase Fic	1	1	1
	Chromosome	FMA85_20770	DUF2559 family protein	4	6	6
	Chromosome	FMA85_21920	type II toxin-antitoxin system YhaV family toxin	1	2	2

Continuation Table S6. Number of specific peptides detected by proteomics analyses for genomic elements in each replicate (A, B, C). Locus tag and replicon of origin are indicated. The peptides unique for particular proteins are used for identification of that protein. Only elements for which peptides have been detected in, at least, two replicates are indicated.

Category	Replicon	NCBI locus tag	Description	A	B	C
	Chromosome	FMA85_22125	mRNA interferase HigB	1	1	2
	pSUH-1	FMA85_25270	type II toxin-antitoxin system toxin CcdB	3	4	3
	pSUH-1	FMA85_25310	type II toxin-antitoxin system VapC family toxin	4	5	6
**TA systems**	pSUH-1	FMA85_25355	type II toxin-antitoxin system death-on-curing family toxin	2	2	3
	pSUH-1	FMA85_25355	type II toxin-antitoxin system death-on-curing family toxin	2	2	3
	pSUH-1	FMA85_25460	type II toxin-antitoxin system RelE/ParE family toxin	1	1	1
	pSUH-1	FMA85_25765	mRNA interferase PemK	4	3	4

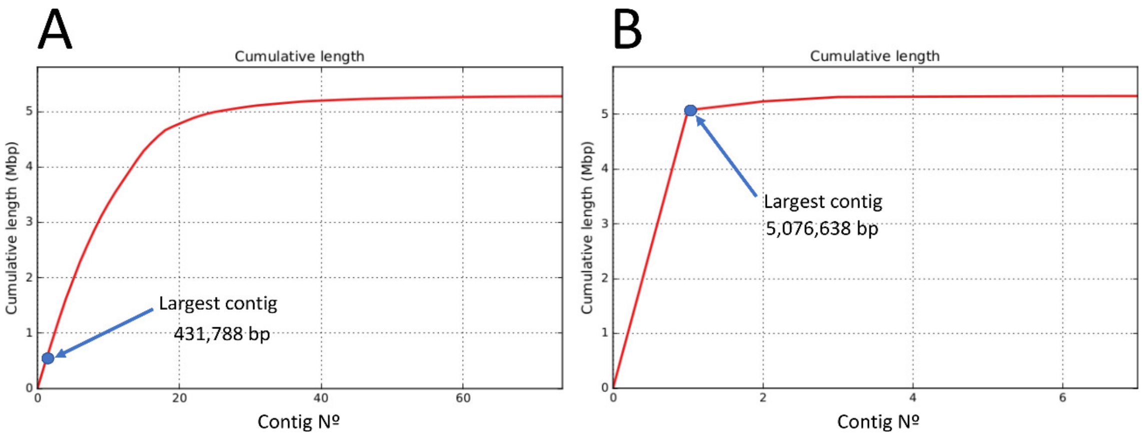

Figure S1. Plots showing the cumulative lengths (Y axis) of genome assembly contigs, ordered from the largest to the smallest (X axis). Marked differences in the sizes and numbers of contigs are shown between, A) only Illumina (short-read) sequence assembly, and B) the assembly combining Illumina short-read with long-read sequence data produced by the MinION sequence platform.
